# Facilitated Peptide Transport *via* the Mucosal Epithelium: Impact on Tolerance Induction

**DOI:** 10.3389/fimmu.2017.00216

**Published:** 2017-03-06

**Authors:** Elisabeth E. Kenngott, Jennifer Pfeil, Ute Hoffmann, Uta Lauer, Anja A. Kühl, Anne Rigby, Anton Pernthaner, Alf Hamann

**Affiliations:** ^1^Experimental Rheumatology, Deutsches Rheuma-Forschungszentrum, Berlin, Germany; ^2^Rheumatology, Department of Gastroenterology, Infectiology and Rheumatology, Charité Universitätsmedizin Berlin, Berlin, Germany; ^3^Experimental Rheumatology, Department of Rheumatology and Clinical Immunology, Charité Universitätsmedizin Berlin, Berlin, Germany; ^4^Medical Department, Division of Gastroenterology, Infectiology and Rheumatology, Research Center ImmunoSciences, Charité Universitätsmedizin Berlin, Berlin, Germany; ^5^The Hopkirk Research Institute, AgResearch Ltd., Grasslands Research Institute, Palmerston North, New Zealand

**Keywords:** oral tolerance, peptide vaccination, targeted delivery, mucosa, regulatory T cells, mucosal uptake

## Abstract

A hallmark of autoimmunity is the breakdown of tolerance and generation of effector responses against self-antigens. Re-establishment of tolerance in autoimmune disorders was always the most desired treatment option; however, despite many efforts, clinical trials have been largely unsuccessful. This also applies to the generation of oral tolerance, which seems to be a default response type of the mucosa-associated lymphoid tissues to harmless antigens. In this study, we report improved efficacy of oral tolerance induction by coupling antigen with the newly identified mucosal carrier peptide 13C. Antigen coupled to 13C is efficiently taken up in the gastrointestinal tract and could be visualized in cells of the lamina propria. Oral, rectal, or nasal treatment effectively induced the proliferation of antigen-specific T cells with some increase in the frequency of regulatory T cells. In a model of delayed-type hypersensitivity, especially intrarectal tolerization treatment resulted in reduced footpad swelling, demonstrating a moderate tolerogenic effect of mucosal treatment with 13C coupled antigen. Coupling of antigens to a transmucosal carrier, therefore, is a promising tool to improve the efficacy of vaccination *via* mucosal surfaces.

## Introduction

Mucosal surfaces are constantly exposed to the external environment and are main entry sites for pathogens. The gut mucosa discriminates between pathogens on the one side and harmless food antigens and commensals on the other side, with antigens from the latter categories rather eliciting ignorance or active tolerance in the immune system than inducing effector mechanism.

Whether a foreign antigen induces immunity or tolerance is determined by intrinsic and conditional factors: the recognition of antigen or its carrier by receptors of the innate immune system, such as toll-like receptors, determines the outcome of the response. Conditions where inflammatory signals are abundant, either in the environment or on purpose by vaccine adjuvants, lead to the generation of strong effector responses. In contrast, antigens that do not stimulate the innate system, such as soluble antigens or peptides, appear to elicit induction of tolerance as a default response. Notably, oral administration of soluble antigens has been shown to induce systemic unresponsiveness and can be mediated by the induction of regulatory cells or by clonal deletion and/or induction of anergy in effector cells, a response designated as oral tolerance (1, 2).

Manipulation of induction of oral tolerance is seen as an attractive means to induce antigen-specific tolerance to prevent autoimmune diseases. However, to date, clinical trials have largely been unsuccessful (3). This might depend on two major issues: first, pro-inflammatory conditions, prevalent in patients affected with autoimmune disease might suppress successful establishment of tolerance. It appears conceivable, however, that concomitant inhibition of inflammatory pathways might overcome this issue in future approaches. Second, vaccination *via* the oral route is of poor efficacy due to poor uptake and rapid degradation of antigen in the gastrointestinal tract, in particular when peptides are used. In the present study, we investigated if coupling of antigenic peptides to a specific cyclic peptide, which facilitates transportation through the gut epithelium, promotes selective uptake, and thereby induction of tolerance.

We recently identified the 13C peptide that increases uptake and transcytosis of peptides and proteins upon mucosal administration (4). The study demonstrated that labeled 13C peptide as well as peptide or protein bound to it is rapidly taken up by subsets of epithelial cells in the intestinal mucosa. These cells display the morphology of goblet cells which already have been implicated in the spontaneous uptake of antigens (5, 6). The constructs were then either transported into lymph and subsequently into systemic circulation, or taken up by CD11c-positive cells, most likely dendritic cells (DCs), of the lamina propria and Peyer’s patches. The 13C peptide, therefore, is considered a promising candidate for the delivery of antigens to the immune compartment for the induction of tolerance *via* the intestinal route.

In the present work, we assessed the tolerogenic potential of mucosal vaccinations using 13C peptide-coupled ovalbumin peptide (OVA323-339: pOVA) and tested T cell responses in the DO11.10 transfer model system (7). Antigen-induced proliferation, induction of Foxp3^+^ Tregs, production of IL-10, TNF, and other cytokines was analyzed in reactive T cells, and the impact of mucosal vaccination in a delayed-type hypersensitivity (DTH) reaction was measured to detect dominant tolerance. We found that oral, rectal, and intranasal administration of 13C-pOVA peptide significantly increased proliferation of antigen-specific T cells. Increased frequencies of regulatory T cells as well as a shift of the ratio of IL-10 to TNF in favor of IL-10 was observed in some conditions. Especially intrarectal application of 13C coupled to pOVA resulted in a reduced footpad swelling in the DTH model. Thus, the use of the described targeting approach might be useful to improve oral tolerization protocols.

## Materials and Methods

### Mice

Female Balb/c mice were purchased from Charles River (Sulzfeld, Germany) and used at 8–10 weeks of age. DO11.10 pOVA-TCR transgenic mice extensively backcrossed (>10 generations) onto Balb/c background were housed in the DRFZ breeding facility. All mice were maintained under specific pathogen-free conditions according to national and institutional guidelines. All experiments were approved by Landesamt für Gesundheit und Soziales (LAGeSo; G0014/14).

### Peptide Synthesis

OVA peptide 323–339 (pOVA: ISQAVHAAHAEINEAGR), 13C-pOVA peptide (CTANSSAQC-βAla-ISQAVHAAHAEINEAGR), and 13Csc-pOVA-peptide (scrambled control; CSNAQATSC-βAla-ISQAVHAAHAEINEAGR) were synthesized in house (Institute for Medical Immunology, Charité Universitätsmedizin Berlin, Germany) as described previously. 13C-FITC and 13Csc-FITC were synthesized by jpt peptides (Berlin, Germany). All 13C or 13Csc constructs were cyclysed using 10% DMSO (v/v) in H_2_O with a pH 8–8.5.

### Cell Preparation

Single cell suspensions were prepared from lymph nodes and spleen. Red blood cells were lysed in lysis buffer [0.01 M KHCO_3_; 0.155 M NH_4_Cl, 0.1 mM ethylenediaminetetraacetic acid (EDTA)] and washed with phosphate buffered saline (PBS) containing 0.2% BSA. CD4^+^ T cells were enriched using anti-CD4 microbeads (clone: RM4-5, Miltenyi Biotec, Bergisch Gladbach, Germany) according to manufacturer’s instructions and sorted using an AutoMACS Pro (Miltenyi Biotec). Cell enrichment was verified by flow cytometric analysis; a frequency of >94% CD4^+^ cells was considered successful. Generally, the frequency of pOVA-specific cells (KJ1.26^+^) was between 70–80% within the CD4^+^ cell pool.

### *In Vitro* Proliferation Assay

For *in vitro* analysis, CD4^+^ cells from DO11.10 mice were labeled with 1 μM CFSE in PBS. The CD4-negative fraction was depleted from the remaining T cells using anti-CD90 microbeads (Miltenyi Biotec) and AutoMACS separation and used as antigen-presenting cells (APCs). APCs were irradiated (30 Gy for 28 min) and cultured with CD4^+^ cells in a final concentration of 2 × 10^6^ cells/ml cRPMI [RPMI 1640 Glutamax medium (Gibco, Paisley, UK) supplemented with 10% fetal calf serum, penicillin (100 U/ml), streptomycin (100 μg/ml), 2-mercaptoethanol (1 mM), sodium pyruvate (1 mM), and HEPES (*N*-2-hydroxyethylpiperazine-*N'*-2-ethanesulfonic acid; 25 mM)] with a ratio of 4:1 in 96-well round-bottom plates. pOVA constructs were added in various concentrations as indicated. Cells were incubated for 4 days at 37°C in a humidified 5% CO_2_ atmosphere. For analysis of proliferation, cells were stained and analyzed by flow cytometry by gating on pOVA-TCR^+^ CD4^+^ cells and calculating the geometrical mean of the fluorescence intensity (GMFI) of the CFSE signal. Fold CFSE dilution was determined [fold CFSE dilution: GMFI (PBS control)/GMFI (sample)].

### *In Vivo* Proliferation Assay

Purified total CD4^+^ CFSE-labeled cells isolated from peripheral lymph nodes and spleens of DO11.10 mice were adoptively transferred i.v. into Balb/c mice (5 × 10^6^ cells/mouse). On the following day, mice were treated with peptide-pOVA constructs (pOVA, 13C-pOVA or13Csc-pOVA) or PBS, either i.v., orally, rectally or nasally as indicated. For oral treatment, mice were fed intragastral with 0.5 mg peptide or equimolar amounts of peptide conjugates in 200 μl PBS using a feeding needle (FST, Heidelberg, Germany). For rectal treatment, mice were injected rectally with 0.5 mg pOVA or equimolar amounts of peptide-conjugates (unless otherwise indicated) in 200 μl PBS using an umbilical catheter with a 0.5 mm × 0.8 mm diameter. For nasal treatment, mice were anesthetized with 100 μl Dexdomitor (0.4 mg/ml, Pfizer, New York, NY, USA) and short isoflourane (Abbott, Chicago, IL, USA) inhalation. After intranasal application of 0.05 mg pOVA or equimolar amounts of peptide conjugates in 20 μl PBS, mice were injected with 100 μl Antisedan (2 mg/ml, Pfizer).

### DTH Reaction

Balb/c mice received a transfer of CD4^+^ cells from DO11.10 donor mice and were tolerized intrarectally as described before in the *in vivo* proliferation assay. On day 8 after cell transfer, the mice received a subcutaneous (tailbase) injection of pOVA (125 μg) in complete Freund’s adjuvans [complete Freund’s adjuvant (CFA); Sigma-Aldrich, St. Louis, MO, USA]. To induce a strong DTH reaction on day 22, the mice were injected into the right footpad with 250 ng of pOVA in 5 μl of incomplete Freund’s adjuvans [incomplete Freund’s adjuvant (IFA), Sigma-Aldrich]. PBS/IFA emulsion was injected into the left footpad as a control. The footpad swelling was determined 24 h after challenge using an Oditest micrometer gauge (Kroeplin Längenmesstechnik, Schlüchtern, Germany).

### Antibodies and Flow Cytometry

The following antibodies and reagents were obtained from eBioscience (San Diego, CA, USA): eFlour 450-conjugated anti-CD4 (RM4-5), PE conjugated anti-IL-10 (JES5-16E3) PE-Cy7 conjugated anti-IFNγ (XMG1.2), PerCP-eFlour 710 conjugated anti-TNFα (MP6-XT22), eFlour 450-conjugated anti-Foxp3 (FJK-16s), and appropriate isotype controls. V500-conjugated anti-CD4 (RM4-5) antibody was purchased from BD Bioscience (Heidelberg, Germany). Cy5-conjugated anti-pOVA-TCR antibody (KJ1.26) and anti-Fcγ-receptor antibody (2.4G2) were produced in house (Deutsches Rheuma-Forschungszentrum Berlin). Total rat IgG was purchased from Dianova (Hamburg, Germany).

Cell surface staining of lymphocytes was performed in the presence of anti-Fcγ-receptor antibody (20 μg/ml). Intracellular Forkhead-box-protein 3 (Foxp3) staining was performed using the anti-mouse Foxp3 staining set (eBioscience, San Diego, CA, USA) according to manufacturer’s instructions.

For cytokine staining, cells were stimulated with phorbol-12-myristate 13-acetate (PMA 10 ng/ml) and ionomycin (500 ng/ml) (Sigma-Aldrich) for 2 h at 37°C in a humidified 5% CO_2_ atmosphere. Brefeldin A (10 μg/ml) (Sigma-Aldrich) was added, and cells were incubated for another 2 h. After surface staining, cells were fixed by incubation with 2% paraformaldehyde (PFA). Intracellular staining was performed in PBS containing 0.5% saponin (Sigma-Aldrich) after 5 min pre-incubation with blocking rat IgG. Flow cytometry was performed using a FACS Canto II (BD Bioscience) and analyzed with Flow Jo software (TreeStar, Ashland, OR, USA).

### Microscopical Analysis

For visualization of peptide uptake, BALB/c mice were anesthetized by intraperitoneal (i.p.) injection of Ketamin/Xylazin solution (200 and 10 mg/kg, respectively; Ketavet^®^, Zoetis, Berlin, Germany; Rompun^®^, Bayer, Leverkusen, Germany) and fluorescein isothiocyanate (FITC) coupled peptide construct were injected into a ligated loop of the large intestine (0.02 μg/μl). A 1.5-cm loop was injected with approximately 100 μl. After 10 min incubation, the tissue was removed and fixed in 2% PFA solution for approximately 16 h. For dehydration, the tissue was incubated in increasing solutions of sucrose (10, 20, and 30%), for 2 days at 4°C. After tissue embedding and freezing, 7 μm cryostat sections were made using the HM 560 Cryotom (Thermo Fischer Scientific, Waltham, MA, USA). Slides were frozen at −20°C until further use.

For immunohistochemistry, the slides were rehydrated in PBS. Washing steps were performed using PBS containing 1% BSA and 0.1% Tween20. The blocking buffer additionally contained 10% rat serum. After incubation with blocking buffer, the sections were stained with anti-CD11c mAb (clone N418; produced in-house) conjugated to AlexaFlour^®^647 and washed twice. Immediately before analysis, the slides were mounted with DAKO mounting medium containing DAPI (4,6-diamino-2-phenylindole; Sigma-Aldrich). The slides were analyzed using a LSM 710 confocal microscope (Carl Zeiss, Jena, Germany), and pictures were taken with the software Zen 2011 (Carl Zeiss MicroImaging GmbH, Göttingen, Germany).

For histopathological analysis in the DTH mouse model, paws were fixed in formalin, bone material was decalcified employing 0.5 M EDTA pH8 and whole paws were embedded in paraffin. Paraffin sections were cut (1–2 μm), dewaxed, and stained histochemically with hematoxylin and eosin (H&E) for overview. H&E stained sections were evaluated for epidermal changes like immune cell infiltration, spongiosis, and scurf as well as for inflammation of the dermis.

### Statistics

Data were analyzed using Prism 5 (GraphPad, La Jolla, CA, USA). Statistical tests include: Mann–Whitney test, followed by Holm–Bonferroni correction for multiple comparisons, multiple comparison analysis using Kolmogorov–Smirnov and D’Agostino and Pearson omnibus test, non-parametric Kruskal–Wallis test followed by Dunn’s post test and One-way ANOVA followed by Tukey’s or Bonferroni’s post test. Statistical analysis of cell proliferation was calculated using the fold-change values. Differences were considered as statistically significant with *p* ≤ 0.05 (*), very significant with *p* ≤ 0.01 (**) and extremely significant with *p* ≤ 0.001 (***) or marked as non-significant (n.s.).

## Results

### Coupling of 13C to pOVA Peptide Does Not Alter Its Recognition *In Vitro* and *In Vivo*

An *in vitro* proliferation assay was employed to verify that modification of pOVA by conjugation to the cyclic 13C peptide does not impair presentation by APCs and recognition by antigen-specific T cells. pOVA or equimolar amounts of 13C-pOVA were tested in a 4-day culture of APCs (CD4^-^CD90^-^) and carboxyfluorescein succinimidyl ester (CFSE)-labeled pOVA-specific CD4^+^ T cells from DO11.10 mice. The gating strategy is available in Supplementary Material (Figures S1A,B in Supplementary Material). We found that T cell proliferation and dose response curves were similar for pOVA and 13C-pOVA (Figure S2A in Supplementary Material). Coupling of 13C to pOVA, therefore, did not change the capacity of the model antigen for T cell activation. This was also verified in *in vivo* experiments. One day prior to the peptide treatment, CD4^+^ cells from DO11.10 donor mice were adoptively transferred into Balb/c mice. Intravenous (i.v.) injection of 5 μg (2.8 nmol) pOVA, equimolar amounts of 13C-pOVA, or of a control peptide with a scrambled amino acid sequence coupled with pOVA (13Csc-pOVA) led to similar levels of T cell proliferation in the spleen, further confirming that pOVA is fully functional when conjugated to either 13C or 13Csc (Figure S2B in Supplementary Material).

### Mucosal Administration of 13C-pOVA Leads to Increased Proliferation of Antigen-Specific Cells

**Figure 1:**
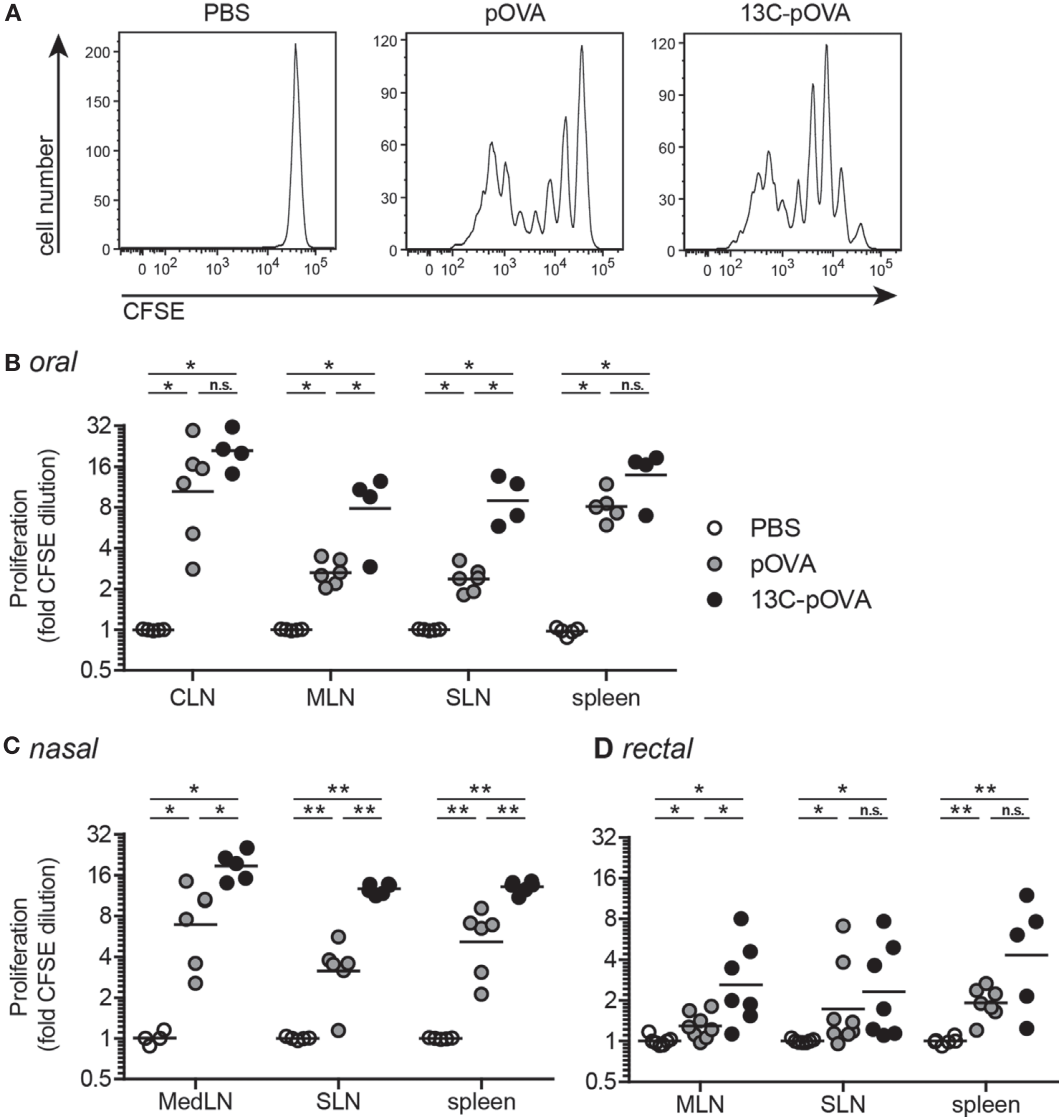
**Coupling 13C to pOVA significantly enhances proliferation of antigen-specific cells upon mucosal application**. Balb/c mice were injected with 5 × 10^6^ CFSE-labeled CD4^+^ cells from DO11.10 donor mice. Twenty-four hours later, mice were treated mucosally with pOVA, 13C-pOVA, or phosphate-buffered saline (PBS) as control. After 3 days, animals were sacrificed and the proliferation of pOVA-TCR^+^CD4^+^ T cells in different organs was analyzed by flow cytometry. **(A)** Representative histograms showing the CFSE dilution in the spleen after oral treatment with PBS, 0.5 mg (0.28 μmol) pOVA, or equimolar amounts of 13C-pOVA. **(B)** T cell proliferation of mice, treated orally with 0.5 mg pOVA or equimolar amounts of 13C-pOVA. **(C)** Mice received 0.05 mg pOVA peptide or equimolar amounts of 13C-pOVA intranasally (nasal). **(D)** Mice were injected intrarectally with 0.5 mg pOVA peptide or equimolar amounts of 13C-pOVA. Pooled data from two to three individual experiments with the Geometric Mean are shown; symbols represent values from individual mice (*n* = 4–8). Statistical testing was performed for each individual organ using the non-parametric Mann–Whitney test and Holm–Bonferroni correction for multiple comparisons. The complete statistical results are shown in Table S1 in Supplementary Material. CLN, cervical lymph nodes; MLN, mesenteric lymph nodes; SLN, subcutaneous lymph nodes; MedLN, mediastinal lymph nodes.

To assess whether coupling of 13C to pOVA improves T cell responses upon administration *via* different mucosal routes, Balb/c mice adoptively transferred with CFSE-labeled pOVA-TCR^+^CD4^+^ T cells received pOVA or equimolar amounts of peptide conjugates by mucosal application. We found that mucosal application of 13C-pOVA resulted in significantly enhanced T cell proliferation when compared to pOVA. To assess whether the facilitated transport is restricted to distinct tissues, we applied the conjugates intragastrically (“oral”), rectally, and nasally. The proliferation of antigen-specific T cells was increased after treatment with 13C-pOVA compared to pOVA alone (Figure 1), albeit the effect differed in strength and occurrence among the immune tissues and delivery routes tested. Intranasal application of 13C-pOVA induced the highest responses overall, with significantly higher proliferation in all three analyzed organs, mediastinal (MedLN) and mesenteric lymph nodes (MLN), and spleen, compared to the PBS-treated group (Figure 1C). Similarly, oral application of 13C-pOVA significantly increased proliferation in the MLN as well as in subcutaneous LN (SLN; pooled axillary, brachial, and inguinal LN) compared to PBS treatment (Figure 1B). Rectal application of 13C-pOVA increased the proliferation especially in the MLN (Figure 1D). We conclude that the use of the 13C peptide as transporter module results in an increase of *in vivo* T cell responses to peptide antigen administered *via* the oral, rectal, and nasal route.

**Figure 2:**
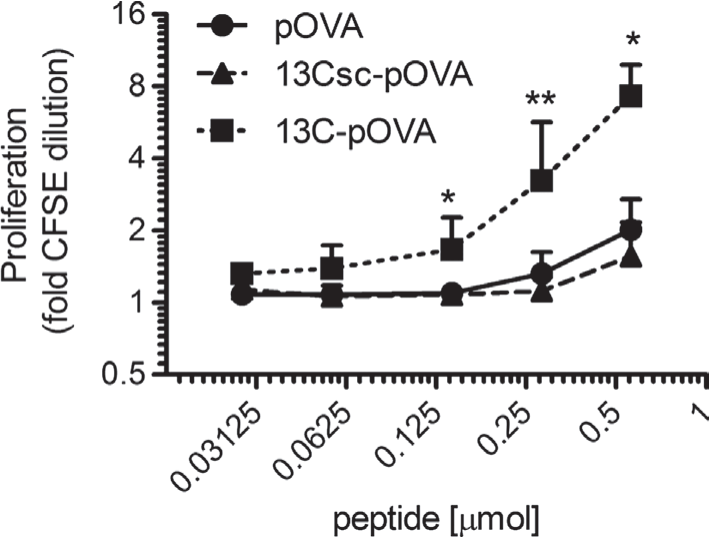
**Intrarectal treatment with 13C-pOVA peptide enhances proliferation of antigen-specific T cells**. Balb/c mice were injected with 5 × 10^6^ CFSE-labeled CD4^+^ T cells from DO11.10 donor mice. Twenty-four hours later, mice were treated intrarectally with indicated amounts of pOVA peptide or equimolar amounts of 13Csc-pOVA or 13C-pOVA. After 3 days, animals were sacrificed and pOVA-TCR^+^CD4^+^ T cells in the mesenteric lymph nodes were analyzed by FACS. Pooled data from one to three individual experiments are shown (*n* = 4–8; Geo. mean ± SD). Statistical testing was performed for each time point using the non-parametric Kruskal–Wallis test.

The antigen-specific T cell response was evaluated in greater detail after intrarectal treatment with pOVA and conjugates of 13C and 13Csc with pOVA. A dose-dependent increase in proliferation was seen for 13C-pOVA which was significantly higher than for pOVA or 13Csc-pOVA, the scrambled control peptide (Figure 2).

**Figure 3:**
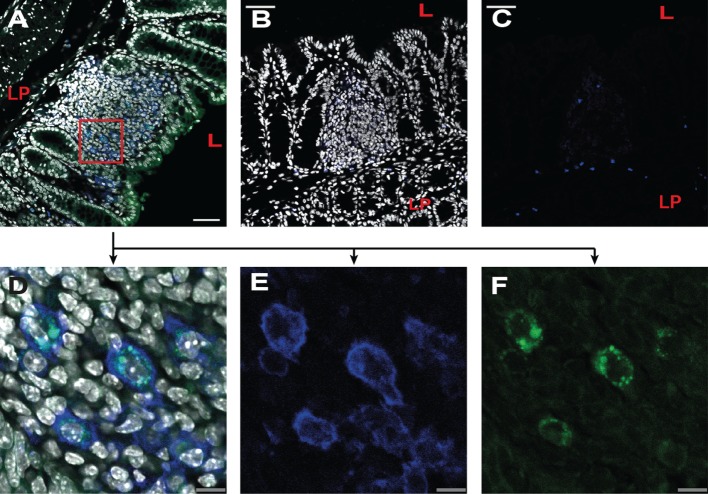
**13C peptide is taken up by epithelial cells and CD11c-positive cells in the large intestine**. Balb/c mice were anesthetized and injected with fluorescein isothiocyanate (FITC)-labeled peptides into a ligated loop of the large intestine. After 10 min, mice were sacrificed and the tissue fixed and frozen. Cryosectioned slides were stained with CD11c-Alexa647 (blue) and DAPI (gray) and analyzed by confocal microscopy. **(A,D–F)** Injection of 13C-FITC (green). **(A)** Lymphoid follicle in the large intestine. Overlay of DAPI, 13C-FITC, and CD11c staining. **(D–F)** Detail of image shown in [**(A)**; red square]. **(B,C)** Injection of the control peptide 13Csc-FITC (green). **(B)** Lymphoid follicle in the large intestine. Overlay of DAPI, 13Csc-FITC and CD11c staining. **(C)** Overlay of 13Csc-FITC and CD11c staining. **(D)** As **(A)**; **(E)** CD11c staining; **(F)** 13C-FITC fluorescence. Pictures are representatives of three individual experiments with similar results. LP, lamina propria; L, lumen; gray, DAPI; blue, CD11c-Alexa647; green, FITC; scale in panels **(A–C)**: 50 μm; scale in panels **(D–F)**: 10 μm.

To visualize the uptake efficiencies in the large intestine, 13C and 13Csc peptides were coupled with FITC and introduced into a ligated loop of the colon (colon ascendens/colon transversum). 13C-FITC was efficiently transported into cells of the lamina propria of the large intestine, already 10 min after injection (Figures 3A,D–F). In contrast, the control conjugate 13Csc-FITC could not be observed in the tissue (Figures 3C,D). Immunofluorescence analyses revealed that epithelial cells had taken up 13C-FITC, but also some of the subjacent CD11c-positive cells in the lamina propria as well as in isolated lymphoid follicles.

### Mucosal Application of 13C-pOVA Leads to a Moderate Induction of Regulatory T Cells and Significantly Decreases the Frequency of Effector T Cells

To analyze whether transporter-mediated mucosal uptake of peptide induces a tolerogenic response as described for some oral immunization protocols [reviewed in Ref. (8)], we analyzed the expression of Foxp3, the master transcription factor of Tregs, as well as the cytokine expression of antigen-specific T cells that became activated upon mucosal application of the peptides.

**Figure 4:**
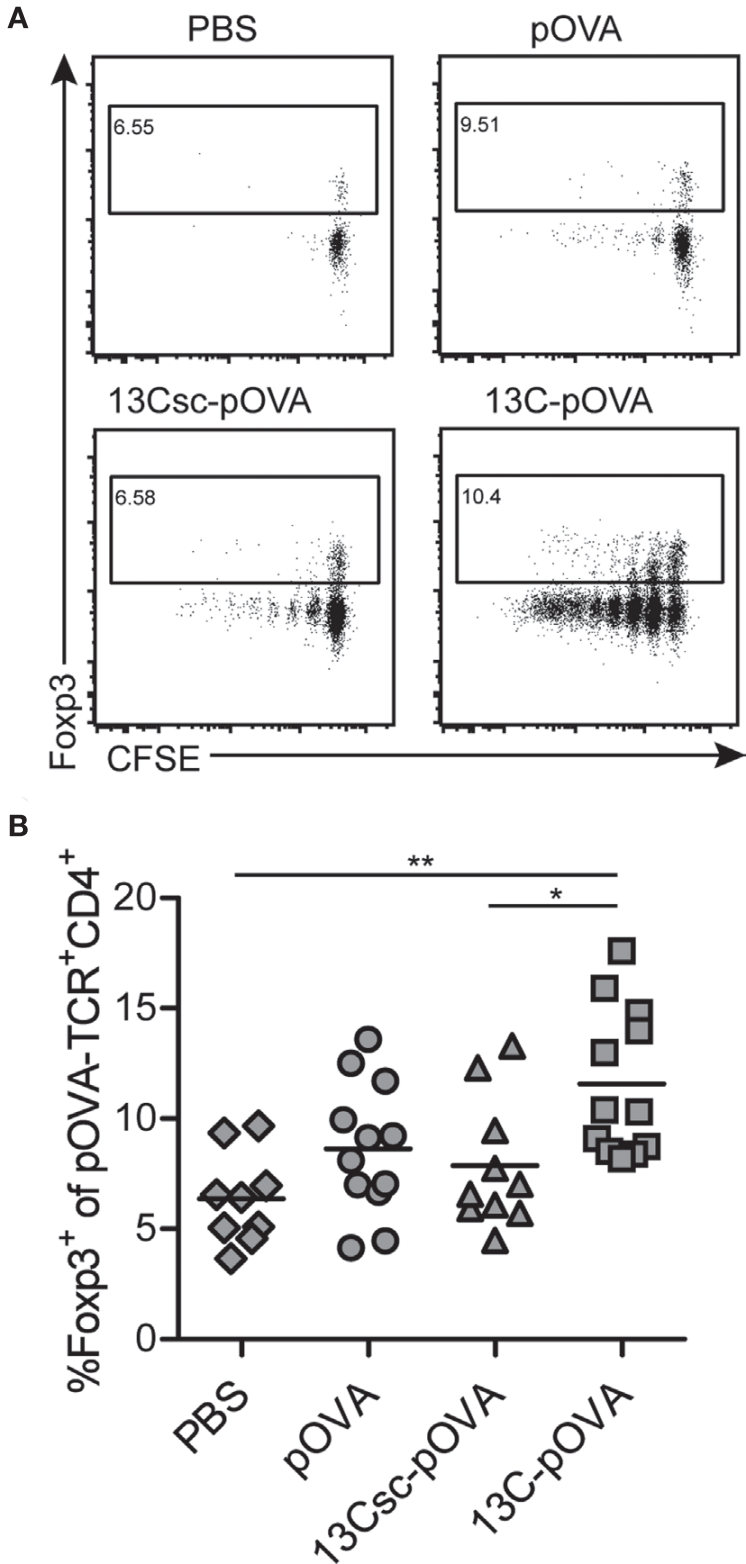
**13C-pOVA treatment slightly increases the frequency of regulatory T cells after intrarectal treatment**. Mice were treated as described in Figure 1 and isolated cells from mesenteric lymph nodes were analyzed by flow cytometry on day 3 after peptide treatment for the expression of Forkhead-box-protein 3 (Foxp3). **(A)** Exemplary FACS dot plots of CFSE dilution and expression of Foxp3 among pOVA-TCR^+^CD4^+^ T cells. **(B)** Frequency of antigen-specific Foxp3^+^ T cells. Pooled data from six individual experiments; symbols represent values from individual mice (*n* = 9–12). Statistical testing was performed using One-way ANOVA (*p* = 0.0017) after confirming normality distribution using a D’Agostino and Pearson omnibus normality test (α = 0.05); statistical symbols represent results from Tukey’s multiple comparison post test.

**Figure 5:**
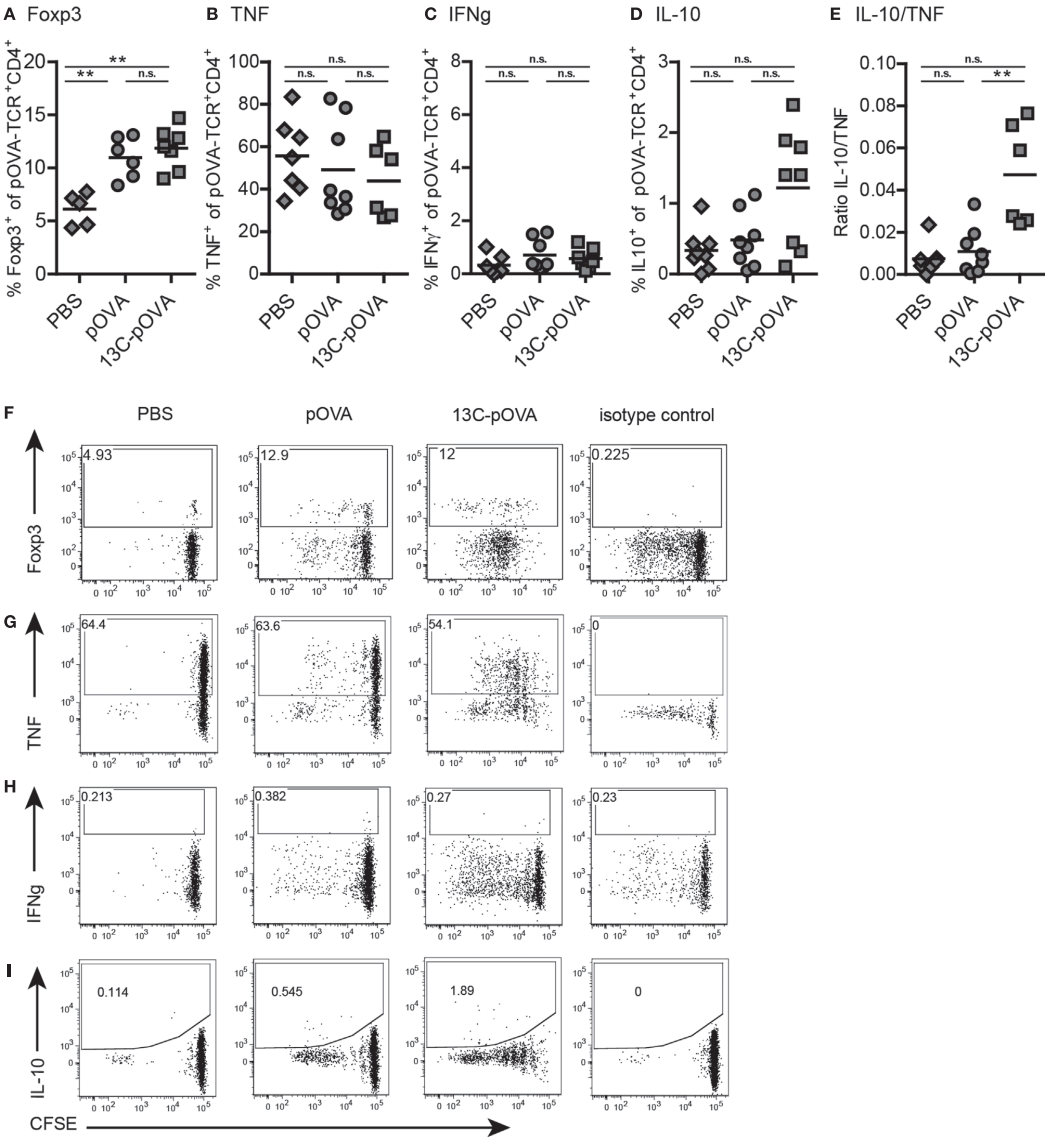
**Changes in production of pro- and anti-inflammatory cytokines**. Mice were treated as described in Figure 1 and isolated cells from mesenteric lymph nodes were analyzed by flow cytometry on day 6 for the expression of Forkhead-box-protein 3 (Foxp3) and various intracellular cytokines. **(A,F)** Foxp3 frequency, **(B,G)** frequency of TNF producing, **(C,H)** frequency of IFNγ producing, and **(D,I)** frequency of IL-10-producing pOVA-TCR^+^CD4^+^ T cells. **(E)** Ratio of T cells expressing IL-10 and TNF. **(A–E)** Pooled data from two to three individual experiments are shown; symbols represent values from individual mice (*n* = 5–8). Statistical testing was performed using the non-parametric Mann–Whitney test and Holm–Bonferroni correction for multiple comparisons. **(F–I)** Representative dot plots.

Intrarectal administration of 13C-pOVA significantly increased frequencies of Foxp3^+^ cells 3 days after application which in the majority are also CD25^+^ (Figures 4A,B; Figure S4 in Supplementary Material). The increase was not due to enhanced deletion of non-Tregs since the frequency of antigen-specific cells remained unchanged compared to control animals (Figure S3 in Supplementary Material). Six days after intrarectal peptide application, frequencies of Foxp3^+^ Tregs were similar in pOVA and 13C-pOVA-treated animals (Figure S5F in Supplementary Material). Contrarily, after oral or nasal treatment, the increase in Foxp3 positive cells in the draining lymph nodes (MLN and MedLN, respectively) was small at day 3 but increased until day 6 (Figures 5A,F; Figures S5A–D in Supplementary Material).

Animals treated with 13C-pOVA rectally exhibited higher frequencies of IL-10 producing T cells compared to treatment with pOVA (Figures 5D,I). As tolerance can also rely on the induction of anergy in antigen-specific cells, we measured the percentage of T cells producing the inflammatory cytokines TNF and IFNγ. Frequencies of TNF^+^ T cells appeared to be reduced upon mucosal application of 13C-pOVA (Figures 5B,G), but these differences were not statistically significant (*p* = 0.23) due to high experimental variability. IFNγ-producing cells were generally very low in numbers and unchanged upon peptide treatment (Figures 5C,H). The calculation of the ratio of IL-10 to TNF-producing cells in individual mice revealed a highly significant bias toward an anti-inflammatory cytokine milieu in 13C-pOVA-treated animals (Figure 5E). We could not observe increased IL-10 frequency after oral treatment in MLN (Figure S6 in Supplementary Material). The IL-10 production in MedLN after nasal treatment could not be measured due to technical reasons.

In none of the mice we detected significant numbers of interferon gamma (IFNγ)-producing T cells under the conditions used here. We conclude that the facilitated mucosal transport of peptide antigen increases the frequency of Foxp3^+^ Tregs. After intrarectal treatment, the cytokine response was shifted toward the anti-inflammatory cytokine IL-10.

### Intrarectal Tolerization with 13C-pOVA Reduces Inflammation in a Model of DTH

**Figure 6:**
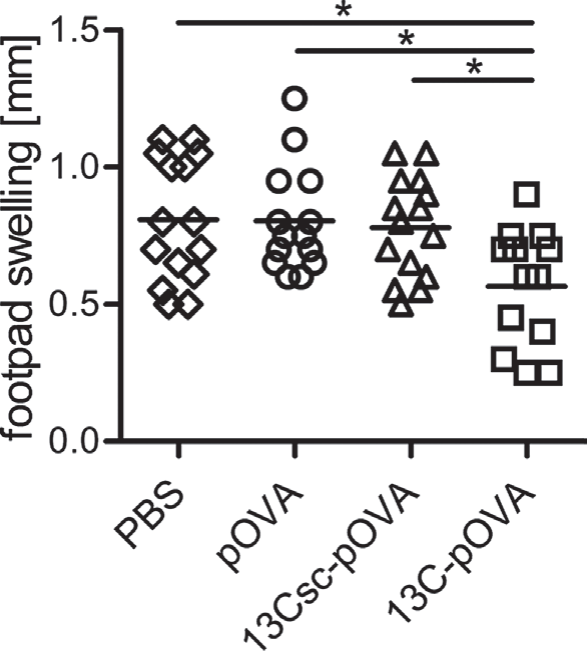
**Reduced footpad swelling in delayed-type hypersensitivity after tolerization with 13C-pOVA peptide**. Balb/c mice were injected with 5 × 10^6^ CFSE-labeled CD4^+^ cells from DO11.10 donor mice. Twenty-four hours later, the mice were tolerized intrarectally with 0.25 mg (0.14 μmol) of pOVA or equimolar amounts of 13C-pOVA or 13Csc-pOVA or phosphate-buffered saline (PBS) as control. On day 8, the mice were immunized by tail-base injection of pOVA/CFA emulsion. On day 22, the mice were injected with pOVA/IFA emulsion into the right footpad (the left footpad received PBS/IFA as control). The following day, footpad swelling was measured. The graph shows the footpad swelling of the IFA/pOVA injected foot compared to the control foot. Pooled data from three individual experiments are shown; symbols represent values from individual mice (*n* = 13–15). Statistical testing was performed using One-way ANOVA (*p* = 0.0082) after confirming normality distribution *via* D’Agostino and Pearson omnibus test (α = 0.05); statistical symbols in graph represent result from Tukey’s multiple comparisons post test.

To further investigate the tolerogenic potency of the 13C-pOVA peptide treatment, the peptide derivatives were tested in a DTH mouse model. Seven days after intrarectal peptide treatment, mice adoptively transferred with DO11.10 cells were injected subcutaneously with pOVA in CFA. Two weeks later, the footpad of the right hind leg was challenged by subcutaneous injection of pOVA in IFA and footpad swelling measured 24 h later. Animals pretreated intrarectally with 13C-pOVA peptide showed a significantly reduced footpad swelling, while DTH was not affected in mice pretreated with pOVA or 13Csc-pOVA (Figure 6). In addition, the frequency of pOVA-TCR^+^CD4^+^ cells in the draining popliteal lymph nodes was analyzed. The foot injected with pOVA/IFA emulsion showed significantly increased infiltration of antigen-specific CD4^+^ T cells compared to PBS, yet frequencies were not affected by the pretreatments (Figure S7 in Supplementary Material). Frequencies of IFNγ and IL-17-producing cells were low among the cells and not changed by the treatment (Figure S8 in Supplementary Material). Additional data from oral and nasal vaccination experiments supported a trend of reduced footpad swelling, increased frequencies of Tregs, and improved histopathology (Figure S9 in Supplementary Material). Taken together, these experiments demonstrated that mucosal pretreatment with pOVA coupled to the transporter 13C is inducing partial tolerance in this animal model.

## Discussion

The gut environment features a number of different tolerogenic mechanisms, including high numbers of Tregs in the lamina propria that contribute to the tendency of mucosal immune reactions that result, in the absence of danger signals, in tolerance (9–11). For a long time, the induction of oral tolerance was seen as an attractive goal for therapeutic application, yet clinical trials have not been successful so far for several reasons (3). Intact proteins have mostly been used for vaccination, however, are difficult to standardize. In contrast, peptides are well-defined and more convenient regarding quality control. For both peptides and proteins, the gastrointestinal immunization route with its limited uptake efficiency and rapid degradation is a major obstacle.

The present study targets the issue of poor uptake of gastrointestinal vaccines by application of a novel transmucosal transporter peptide that was previously found to carry cargo through the mucosal barrier. The 13C peptide, a nine amino acid long cyclic peptide facilitated mucosal uptake of coupled molecules by, as of yet, unknown mechanisms (4). We here studied the impact of this strategy on vaccination efficacy and tolerance induction in the DO11.10/OVA323-339 peptide (pOVA) model. Oral, rectal, and nasal application of 13C coupled to pOVA significantly increased *in vivo* proliferation of antigen-specific T cells compared to application of pOVA in at least some lymphoid tissues. This indicates that 13C-pOVA is taken up *via* the mucosal surfaces more efficiently than non-coupled pOVA, and functionally intact pOVA is delivered to and presented by APC. Consistent with the concept of shared properties of mucosal surfaces and a common mucosa-associated lymphoid tissue, immunization *via* the oral, nasal, and rectal route all were able to activate the pOVA-specific T cells in draining and systemic lymphoid tissues, albeit with quantitative and qualitative differences. Nasal peptide application proved to be most efficient in inducing T cell proliferation. A superiority of the nasal route for T cell priming has also been observed for protein treatment (8, 12). However, in the DTH model studied here, an equal or better tolerogenic efficacy of nasal application could not be confirmed.

Microscopic analysis revealed that the fluorochrome-labeled 13C transporter is taken up while the scrambled control peptide largely remains in the lumen of the large intestine, as already shown previously for the small intestine (4). Uptake is first observed in a subset of epithelial cells that have the morphological features of goblet cells, notably in the small intestine (4), but the conjugate also becomes detectable in mucosal CD11c^+^ cells, which mainly consist of DCs (Figure 3). The proportion of the conjugates remaining in DCs and the proportion being transported *via* lymph to the systemic circulation could not been determined with the present technique. However, the proliferation of antigen-specific cells on day 3 after treatment was comparable in all analyzed lymphoid tissues and was not restricted to the draining LN. This indicates that a major part of the transported peptide is delivered intact into circulation and not only presented on mucosal APC. It can be assumed that the increased proliferation of T cells in mice treated with 13C-pOVA is a consequence of a synergistic effect of faster delivery of the peptide itself throughout the body and direct uptake of the peptides by APC at the site of application. Whether or not migration of peptide-loaded DCs from the mucosa into the draining lymph node contributes to the systemic dissemination of T cell-activating potential, and whether or not a more rapid initial response can be observed at draining sites remains to be determined.

To our surprise we noticed that also some native pOVA was able to cross the mucosal surfaces and elicit T cell responses in mucosal as well as remote lymphoid tissues such as spleen or SLN. Thus, a small fraction of antigenic peptides might naturally access the circulation in intact form; comparing the level of responses to peptide given by i.v. injection versus mucosal application, this fraction can be estimated to be less than 1%. Nevertheless, coupling of pOVA to 13C peptide strongly improved the uptake.

It has been reported that feeding of ovalbumin protein in high doses leads to strong proliferation of the transferred antigen-specific T cells; however, the cells become anergic or undergo activation induced cell death (AICD) (13–15). In contrast, low-dose treatment with ovalbumin protein results in the induction of antigen-specific Tregs and, accordingly, this pathway has been seen as the predominant mechanism of low-dose tolerance (13, 14). However, this view has been challenged by other work, showing that prolonged high-dose treatment can also increase the number of Foxp3^+^ Tregs (16).

The treatment with 0.25 mg (0.14 μmol) pOVA peptide used here is equivalent to approximately 6 mg ovalbumin protein, i.e., a rather low-dose range. Accordingly, a significant increase in the frequency of Foxp3^+^ Tregs compared to the non-immunized animals was recorded, albeit the effect was not as pronounced as in other studies of oral immunization (16). However, despite strong T cell proliferation, indicated by loss of CFSE staining, the frequencies of transgenic T cells had only slightly increased at day 3 (Figure S3 in Supplementary Material). Thus, a significant part of the proliferating cells appears to undergo AICD. While only a minor, non-significant reduction in TNF-producing effector cells was observed upon immunization with peptides, 13C-pOVA induced an increase in IL-10 producing T cells and hence in the ratio IL-10/TNF, suggesting that the peptide immunization *via* the mucosal route increased IL-10-mediated regulatory pathways.

Both IL-10 producing T cells (“TR1”) and Foxp3^+^, Tregs have been implicated in the suppression of inflammation in DTH or other models (17, 18). Indeed, in a DTH model, mucosal application of 13C-pOVA, but not pOVA, resulted in a significant, yet only partial suppression of footpad swelling upon rectal vaccination, and a trend to reduced inflammation upon oral and nasal vaccination (Figure S9 in Supplementary Material). Mucosal vaccination with 13C-pOVA in this model resulted consistently in increased frequencies of Tregs, while the effects on cytokines were less clear after immunization and challenge. A definite analysis of the pathways causing the partial suppression of the DTH requires blocking/deletion experiments, which was beyond the scope of the present study. In previous studies, tolerization in the DTH model was achieved by feeding high doses of ovalbumin protein (18). At the peptide dose applied here, only pOVA coupled to 13C was able to significantly modulate the DTH response. Using this model of a strongly inflammatory disease, it can be concluded that the enhanced uptake of the transporter-coupled antigenic peptide led to more vigorous T cell proliferation, without increased effector cell frequencies, and some increase in antigen-specific Tregs. However, it has to be emphasized that the delivery of antigenic peptide by the mucosal route by itself is not as effective in inducing a robust tolerance as might have been expected. Whether this can be improved by altering conditions (e.g., repeated vaccination) and whether a clear preference for one of the mucosal application routes can be secured, remains to be shown.

We have previously demonstrated that the 13C peptide is also able to carry large proteins in intact form through the mucosal barrier (4). It will be interesting to test whether tolerization with intact proteins using this targeting pathway leads to different results compared with high-dose oral protein application, where most likely only peptides reach the circulation. The ability to deliver macromolecules *via* the mucosal barrier by virtue of the 13C peptide also provides novel opportunities of combining antigens with either tolerogenic or immunogenic adjuvant components, e.g., by coupling additionally either immunosuppressive or stimulatory cytokines to the antigen-transporter complex. Thus, the carrier peptide 13C might become a valuable component in a toolbox of functional units to be used for engineering novel immunomodulatory agents.

## Author Contributions

EK, JP, UH, and AH conceived and designed the experiments. EK, JP, AK, and UL performed the experiments. EK, JP, and AK analyzed the data. EK, AP, and AH wrote and edited the manuscript.

## Conflict of Interest Statement

The authors declare that the research was conducted in the absence of any commercial or financial relationships that could be construed as a potential conflict of interest.
