# Supplementary material for: Facilitated Peptide Transport via the Mucosal Epithelium: Impact on Tolerance Induction
Source: Front Immunol. 2017 Mar 6;8:216. doi: 10.3389/fimmu.2017.00216 (PMC5337492; doi:10.3389/fimmu.2017.00216)
Supplement: Supplementary file 1 [file Data_Sheet_1.PDF]

Supplementary Material

Facilitated peptide transport via the mucosal epithelium: impact on tolerance induction

Elisabeth E. Kenngott[1,2], Jennifer Pfeil[1, 3], Ute Hoffmann[1], Uta Lauer[1], Anja A. Kühl[4], Anne Rigby[3], Anton Pernthaner[5], Alf Hamann[1,3] *

[1]Experimental Rheumatology, Deutsches Rheuma-Forschungszentrum, Berlin, Germany.

[2]Rheumatology, Department of Gastroenterology, Infectiology and Rheumatology, Charité Universitätsmedizin, Berlin, Germany.

[3]Experimental Rheumatology, Department of Rheumatology and Clinical Immunology, Charité Universitätsmedizin, Berlin, Germany

[4]Medical Department, Division of Gastroenterology, Infectiology and Rheumatology/ Research Center ImmunoSciences, Charité Universitätsmedizin Berlin, Germany

[5]The Hopkirk Research Institute, AgResearch Ltd. Grasslands Research, Palmerston North, New Zealand

* Correspondence:
Alf Hamann
hamann@drfz.de

1. Supplementary Figures and Tables

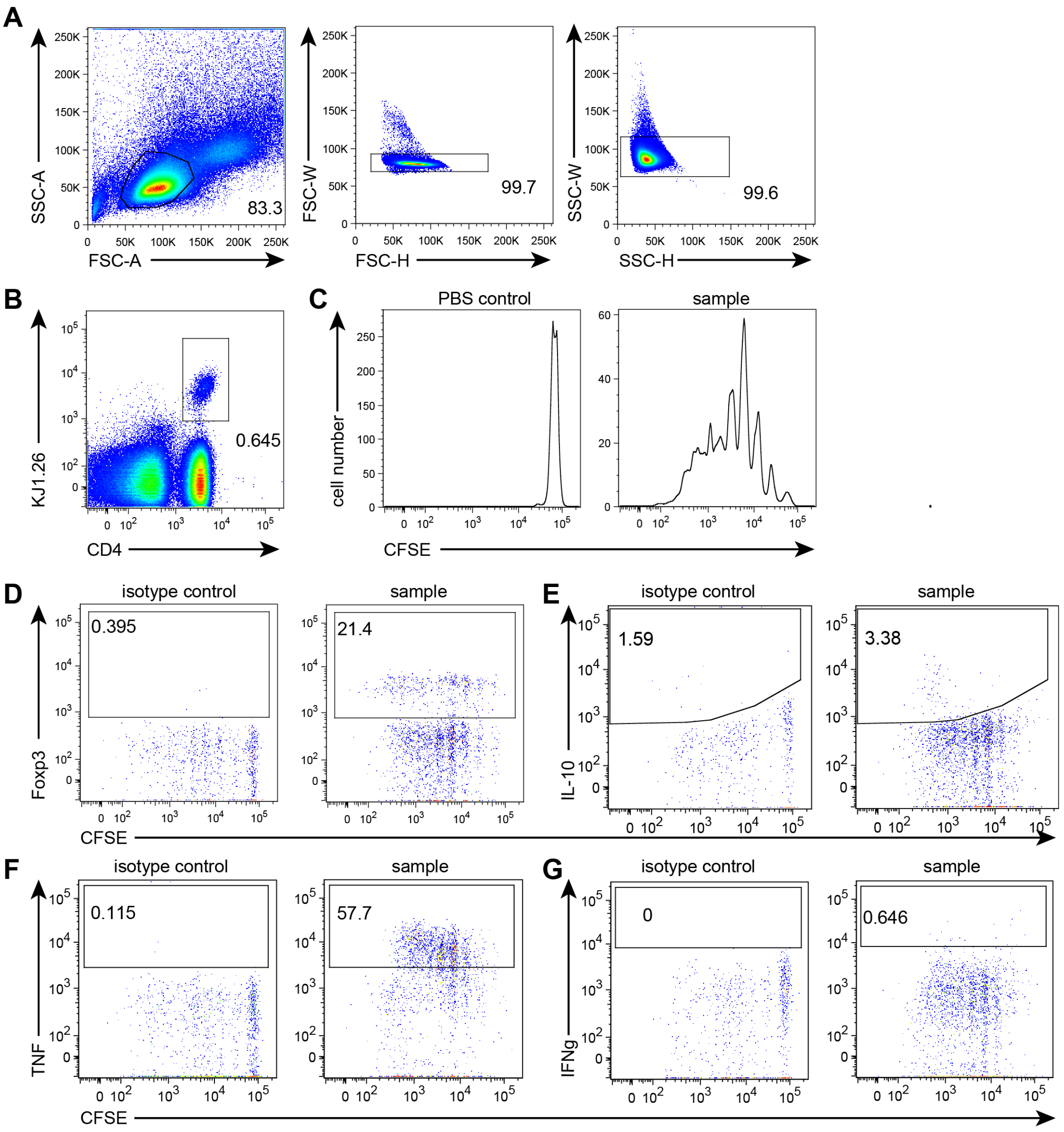

Supplementary Figure 1. Gating strategy after flow cytometric analysis. **(A, B)** Cells were gated on live lymphocytes using the forwards and sideward scatter plot (FSC-A vs. SSC-A). Doublets were excluded first in the forwards, (FSC-H vs. FSC-W) and then in the sideward (SSC-H vs. SSC-W) scatter channel. The remaining cells were then gated on $CD4^{+}$ $KJ1.26^{+}$ cells for analysis of the pOVA-TCR^{+} $CD4^{+}$ T cells. (**C**) The proliferation was analyzed calculating the Geometric Mean (Geo. Mean). Depicted are a PBS control lacking proliferation and a proliferated sample. (**D-G**) Exemplary dot plots of Foxp3 and intracellular cytokine stainings. Gating on intracellular markers was controlled by isotype controls. (**A-F**) Exemplary dot plots and histograms from in vivo proliferation analysis six days after intra nasal tolerization, the exemplary sample shows the analysis after treatment with 13C-pOVA.

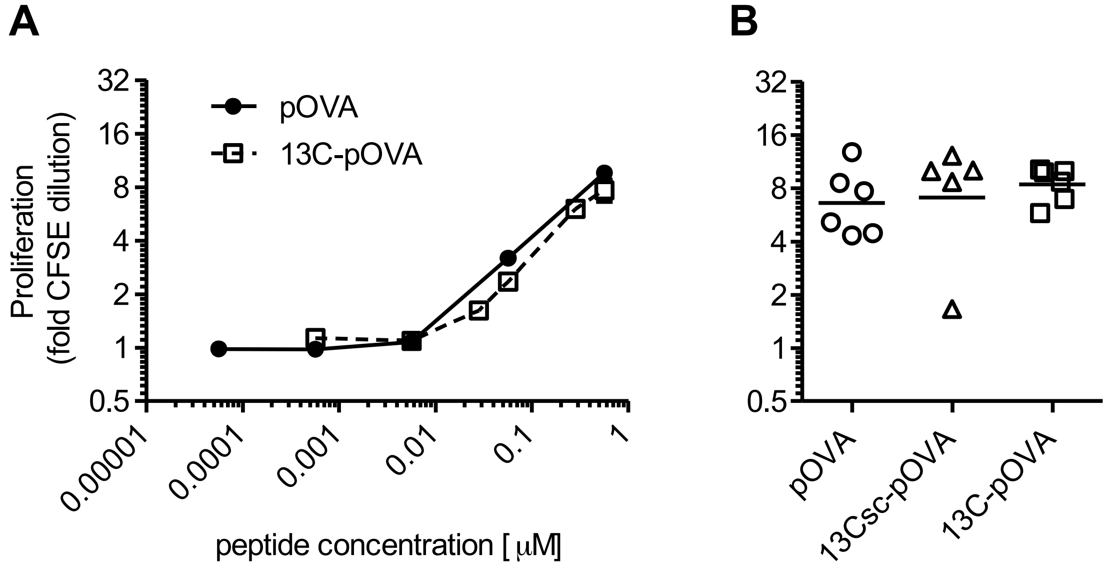

Supplementary Figure 2. Coupling 13C peptide to pOVA peptide does not alter its recognition in vitro and in vivo. (**A**) CFSE-labeled $CD4^+$ T cells from DO11.10 mice were cultured with $CD4^-$ $CD90^+$ antigen-presenting cells in presence of either pOVA or equimolar amounts of 13C-pOVA for four days (1 µM pOVA ≙ 1.77 µg pOVA /ml). Cells were then stained and analyzed by flow cytometry. For analysis of proliferation the fold CFSE dilution was calculated (fold CFSE dilution: GMFI (PBS control)/GMFI (sample). Data from one of three experiments with similar results is shown. (*n*=3; mean ± SD). (**B**) Balb/c mice were injected with 5 x 10^6 CFSE-labeled $CD4^+$ cells from DO11.10 donor mice. 24 h later, 5 µg pOVA peptide (≙ 2.8 nmol) or equimolar amounts of 13C-pOVA or 13Csc-pOVA peptide were injected i.v.. After three days, mice were sacrificed and the proliferation of pOVA-TCR^+CD4^+ T cells in the spleen was analyzed by flow cytometry. Pooled data from two individual experiments is shown; Symbols represent values from individual mice (*n*=5-6).

Supplementary Table 1. Statistical analysis of Figure 1. The Table shows the p-Values obtained in the nonparametrical Mann-Whitney test, the p-Ranking within the group and the significance after Holm-Bonferroni correction using a significance level of either 5% (*) or 1% (**).

**oral**				p-Value of Mann Whitney test	p-Ranking	Significance after Holm-Bonferroni correction
CLN	PBS	vs.	pOVA	0.0043	1	*
	PBS	vs.	13C-pOVA	0.0159	2	*
	pOVA	vs.	13C-pOVA	0.1714	3	n.s.
MLN	PBS	vs.	pOVA	0.0043	1	*
	PBS	vs.	13C-pOVA	0.0159	2	*
	pOVA	vs.	13C-pOVA	0.0381	3	*
SLN	PBS	vs.	pOVA	0.0043	1	*
	PBS	vs.	13C-pOVA	0.0159	3	*
	pOVA	vs.	13C-pOVA	0.0095	2	*
spleen	PBS	vs.	pOVA	0.0079	1	*
	PBS	vs.	13C-pOVA	0.0159	2	*
	pOVA	vs.	13C-pOVA	0.1905	3	n.s.

**nasal**						
MedLN	PBS	vs.	pOVA	0.0139	2	*
	PBS	vs.	13C-pOVA	0.0195	3	*
	pOVA	vs.	13C-pOVA	0.0087	1	*
SLN	PBS	vs.	pOVA	0.0043		**
	PBS	vs.	13C-pOVA	0.0043		**
	pOVA	vs.	13C-pOVA	0.0022	1	**
spleen	PBS	vs.	pOVA	0.0043		**
	PBS	vs.	13C-pOVA	0.0043		**
	pOVA	vs.	13C-pOVA	0.0022	1	**

**rectal**						
MLN	PBS	vs.	pOVA	0.0128	2	*
	PBS	vs.	13C-pOVA	0.0033	1	*
	pOVA	vs.	13C-pOVA	0.014	3	*
SLN	PBS	vs.	pOVA	0.0175	2	*
	PBS	vs.	13C-pOVA	0.0021	1	*
	pOVA	vs.	13C-pOVA	0.5358	3	n.s.
spleen	PBS	vs.	pOVA	0.0012	1	**
	PBS	vs.	13C-pOVA	0.0043	2	**
	pOVA	vs.	13C-pOVA	0.202	3	n.s.

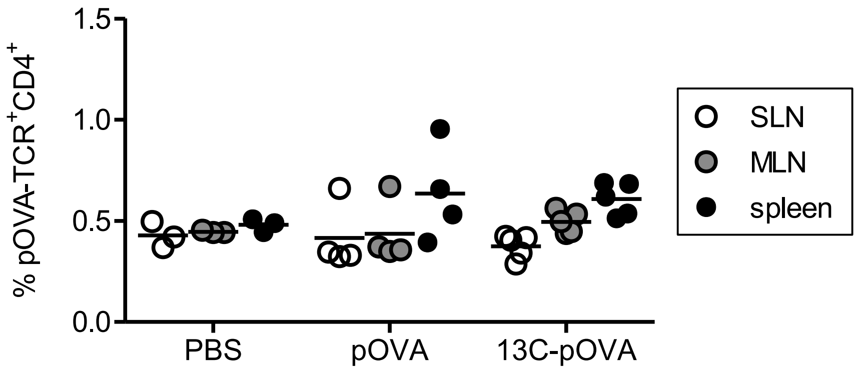

Supplementary Figure 3. The frequency of antigen-specific T cells is unchanged in animals treated with antigenic peptide compared to control animals. Frequency of pOVA-TCR$^+$CD4$^+$ T cells in MLN three days after intrarectal treatment with the respective peptides. Result from one exemplary experiment out of three (*n*=3-5; SLN = subcutaneous lymph nodes; MLN = mesenteric lymph nodes).

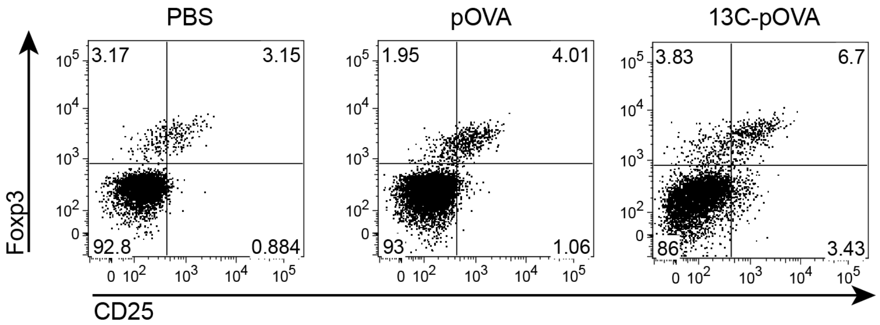

Supplementary Figure 4. The majority of pOVA-TCR$^+$CD4$^+$CD25$^+$ cells are positive for Foxp3. Representative dot plots from flow cytometric analysis of cells in MLN three days after intrarectal treatment with the respective peptides. Live cells were gated on CD4$^+$ and pOVA-TCR$^+$ cells as shown in Supplementary Figure 1.

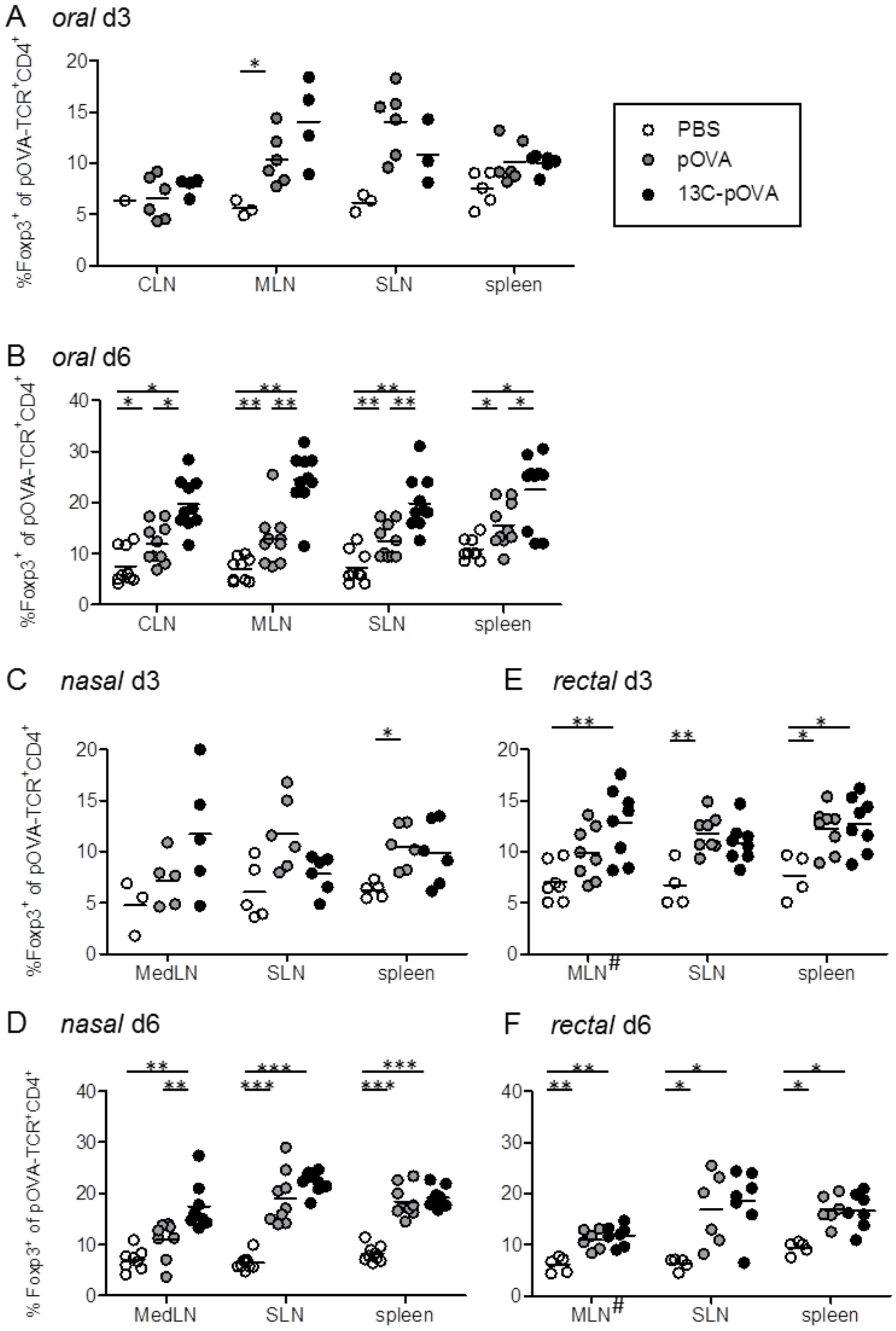

Supplementary Figure 5. Increased Foxp3 frequency after mucosal treatment with 13C-pOVA. Balb/c mice received a transfer of pOVA-TCR$^+$CD4$^+$ T cells and 24 h later were subjected to either oral (**A, B**) nasal (**C, D**) or rectal (**E, F**) treatment with the respective peptides. The Foxp3 frequency was analyzed as described before either at day three (**A, C, D**) or at day six after peptide treatment (**B, E, F**). Pooled data from two to three individual experiments (*n*=4-11). (#) These data in E and F are also shown in Figures 4 and 5, respectively. Statistical testing was performed using the nonparametric Mann Whitney test and the Holm-Bonferroni correction of multiple comparisons (CLN = cervical lymph nodes; MLN = mesenteric lymph nodes; SLN = subcutaneous lymph nodes; MedLN = Mediastinal lymph nodes).

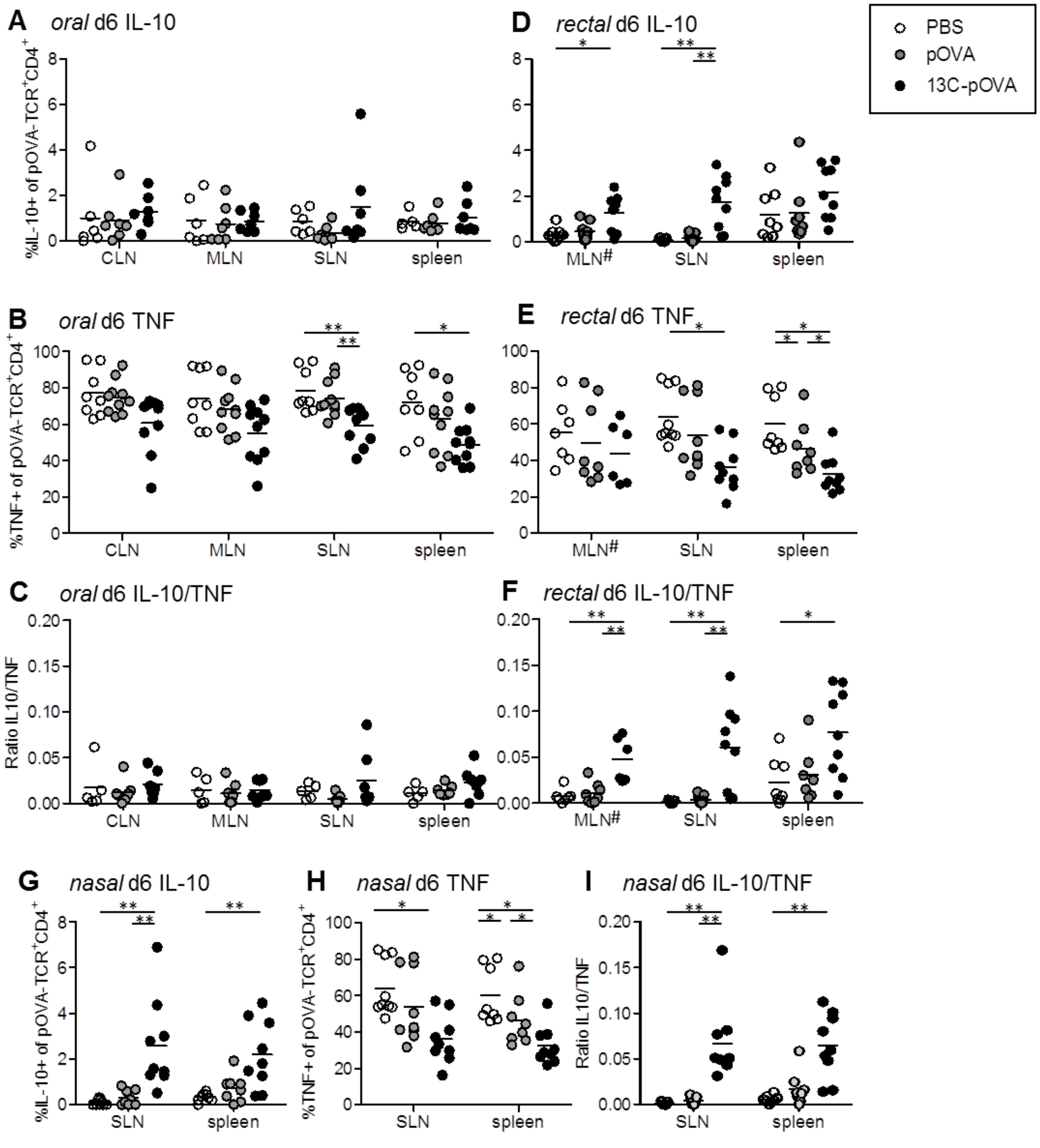

Supplementary Figure 6. Cytokine expression of antigen-specific T cells after mucosal treatment with 13C-pOVA. Balb/c mice received a transfer of pOVA-TCR^{+}CD4^{+} T cells and 24 h later were subjected to either oral (**A-C**), rectal (**D-F**) or nasal (**G-I**) treatment with the respective peptides. Cytokine expression was measured by flow cytometry at day six. (**A, D, G**) Expression of IL-10. (**B, E, H**) Expression of TNF. (**C, F, I**) Ratio of IL10/TNF. Pooled data from two to three individual experiments (*n*=4-11). (#) These data in D, E and F are also shown in Figure 5. Statistical testing was performed using the nonparametric Mann Whitney test and the Holm-Bonferroni correction of multiple comparisons (CLN = cervical lymph nodes; MLN = mesenteric lymph nodes; SLN = subcutaneous lymph nodes).

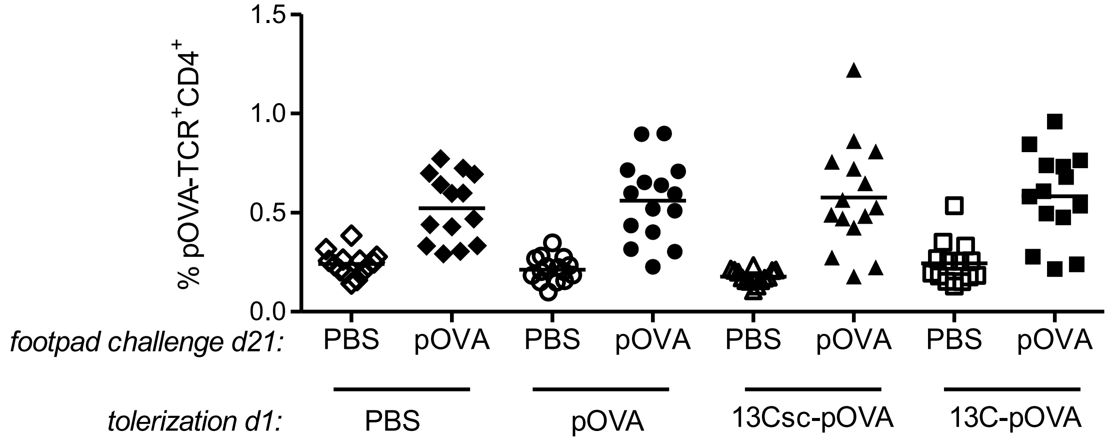

Supplementary Figure 7. Frequency of antigen-specific cells in the draining lymph node is not altered by prior mucosal peptide treatment. Mice were tolerized with the respective peptides intra rectally and underwent DTH by challenging the footpad by IFA/pOVA or IFA/PBS injection. The popliteal lymph node was excised, and the frequency of pOVA-TCR$^+$CD4$^+$ T cells was determined by flow cytometry. Statistical testing was performed using One-way ANOVA: $p<0.0001$ after confirming normality distribution via D'Agostino and Pearson omnibus test ($\alpha=0.05$); Bonferroni's multiple comparison post test revealed statistical differences between the PBS vs. pOVA- footpad challenge in all groups ($p<0.001$) but no statistical difference between the different tolerization groups.

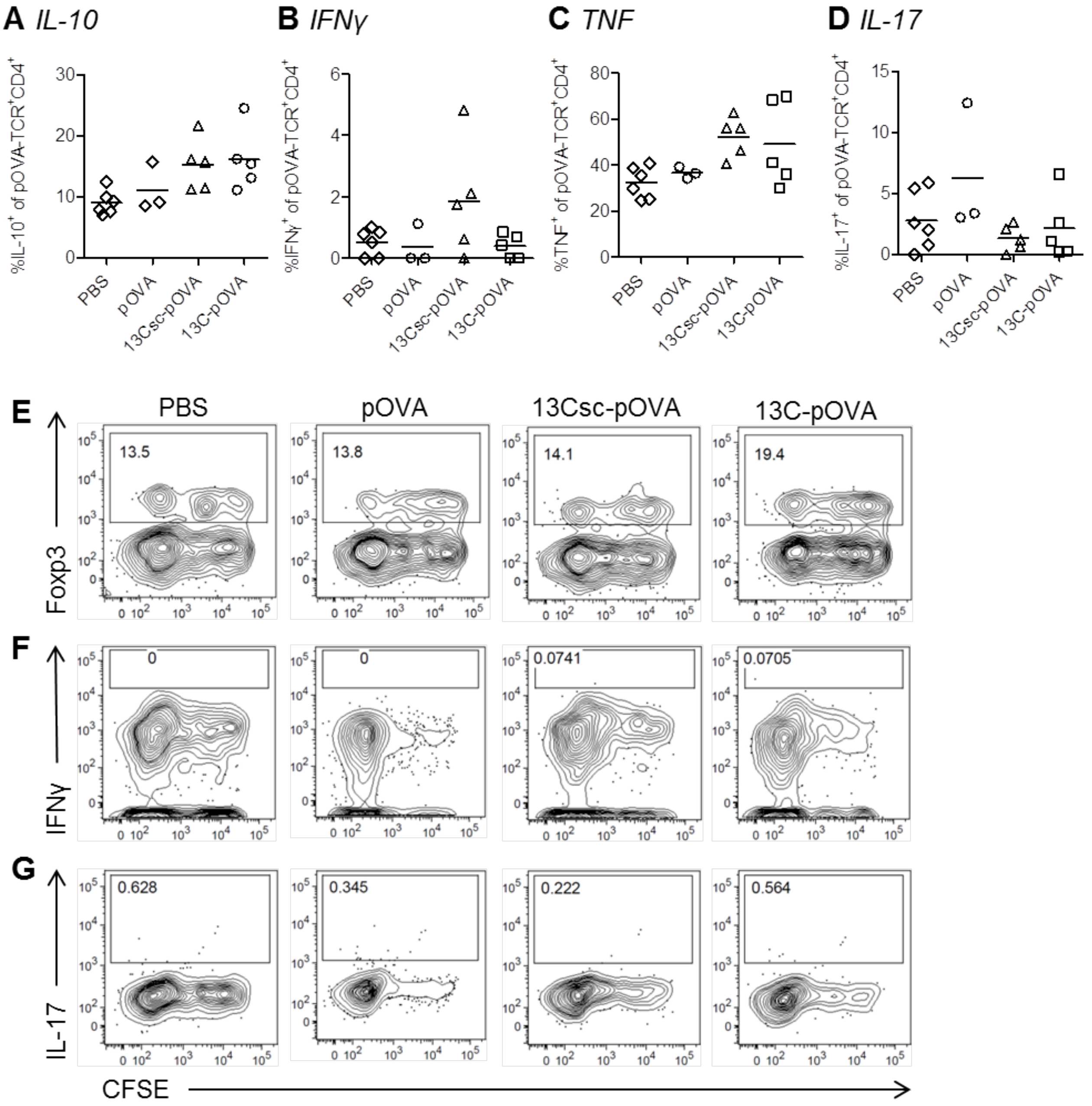

Supplementary Figure 8. Antigen-specific T cells do not produce IFNγ or IL-17 24h after challenge. Mice were tolerized with the respective peptides intra rectally and underwent DTH by challenging the footpad by IFA/pOVA or IFA/PBS injection. The popLN and MLN were excised and the frequencies of Foxp3-expressing or cytokine producing pOVA-TCR^{+}CD4^{+} T cells were determined by flow cytometry. (**A-D**) Expression of cytokines in the popLN of the foot challenged with IFA/pOVA. Examplary data from one experiment (*n*= 3-6). (**E-F**) Examplary dot plots of the expression of Foxp3, IFNγ and IL-17 in the MLN.

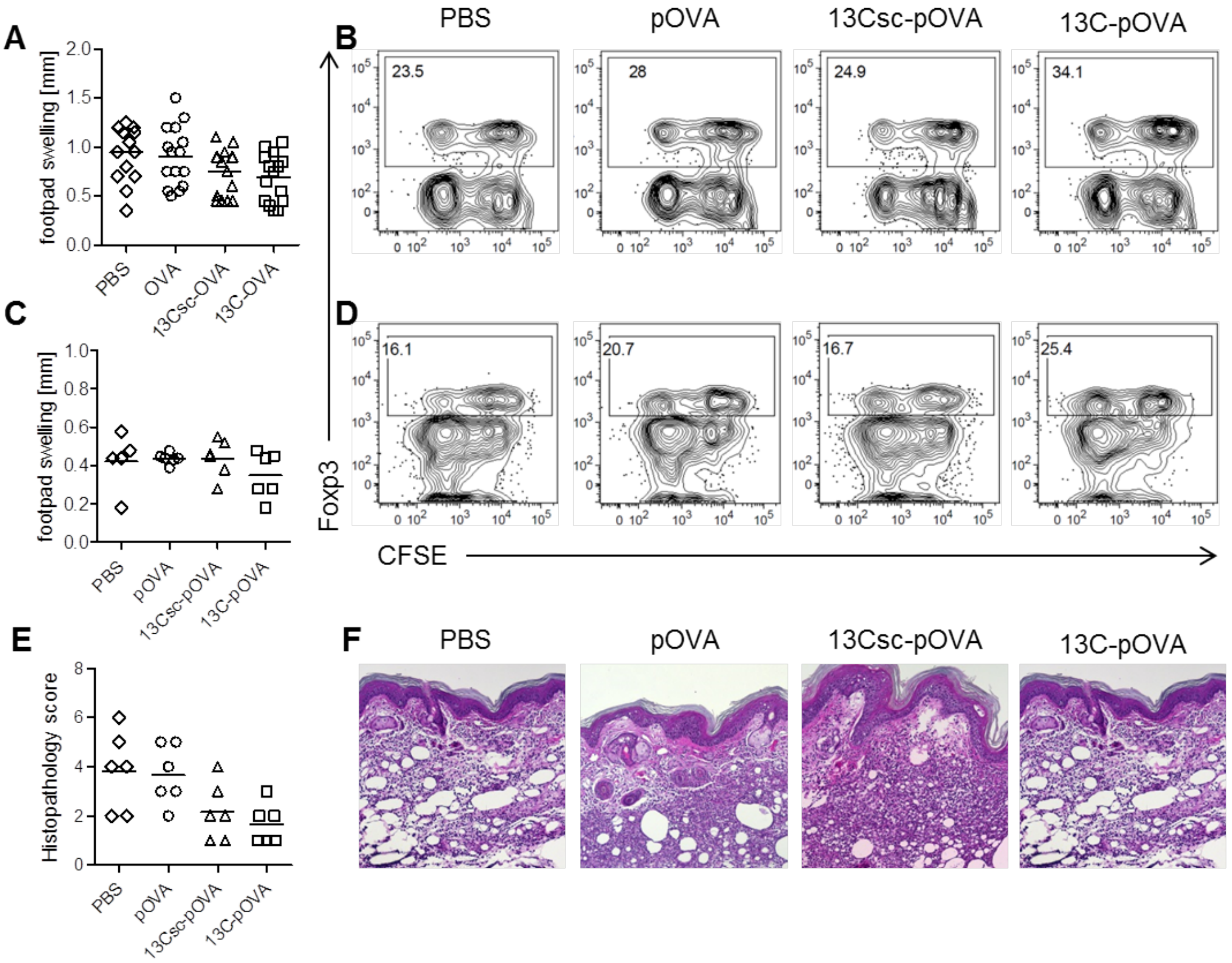

Supplementary Figure 9. Nasal or oral peptide treatment prior to DTH challenge leads to a mild tolerogenic effect. (**A**) Balb/c mice were injected with 5 x 10^6 CFSE-labeled $CD4^+$ cells from DO11.10 donor mice. 24 h later the mice were tolerized intranasally with 0.05 mg of pOVA or equimolar amounts of 13C-pOVA or 13Csc-pOVA or PBS as control. On day eight, the mice were immunized by tailbase injection of pOVA/CFA emulsion. On day 22, the mice were injected with pOVA/IFA emulsion into the right footpad (the left footpad received PBS/IFA as control). The following day, footpad swelling was measured. The graph shows the footpad swelling of the IFA/pOVA injected foot compared to the control foot. Pooled data from three individual experiments is shown; symbols represent values from individual mice (n=13-15). Statistical testing was performed using One-way ANOVA (p=0.0341) after confirming normality distribution via D'Agostino and Pearson omnibus test (α=0.05). (**B**) Exemplary dotplots showing Foxp3 expression and CFSE dilution of antigen-specific T cells in axillary lymph nodes after nasal tolerization. (**C-F**) Mice were treated orally with 0.5 mg pOVA or equimolar amounts of 13C-pOVA or 13Csc-pOVA or PBS as control and were otherwise treated as in A. Data from one exemplary experiment is shown (n=6). (**C**) Footpad swelling of IFA/pOVA injected foot compared to the control foot. (**D**) Exemplary dotplots showing Foxp3 expression and CFSE dilution of antigen-specific T cells in MLN after oral tolerization. (**E**) Histopathological score of challenged foot after oral peptide tolerization. Statistical testing was performed using the Kruskal-Wallis Test (p=0.0217). (**F**) Examplary histochemical images of IFA/pOVA treated paws.
